# A phenomenological study on experiences of cancer stigma amongst selected people living with cancer in rural and urban Zimbabwe

**DOI:** 10.12688/aasopenres.13282.1

**Published:** 2021-10-07

**Authors:** Enock Mandizadza, Stanzia Moyo

**Affiliations:** 1 Community and Social Development, University of Zimbabwe, Harare, Harare, 263, Zimbabwe; 2 Demography Settlement and Development, University of Zimbabwe, Harare, Harare, 263, Zimbabwe

**Keywords:** cancer, health research, qualitative research, self-stigma, enacted stigma, phenomenology, Zimbabwe

## Abstract

**Background: **Cancer is a highly stigmatized illness associated with profound adverse impact on communities, families and diagnosed individuals. Notwithstanding extensive theorizing since Erving Goffman’s classical contributions, health stigma is well explicated in context-specific and situated analysis. The current study explored the manifestations of self and enacted stigma among 20 selected people diagnosed with cancer from rural and urban Zimbabwe, who sought quaternary level of health care services in the capital, Harare.

**Methods: **Phenomenological methodology was enlisted to capture intimate expressions of stigma as expressed about, and by people diagnosed with cancer. Data collection methods used includes semi-structured interviews, key informant interviews and focus group discussions. A semi-structured in-depth interview guide, focus group discussion guide and a key informant interview guide were the tools used to collect the data.

**Results: **The study identified five themes of stigma, indicating pronounced, complex and multiple catalogues of stigma embedded in the existing socio-cultural milieu.

**Conclusions: **This study stands to offer invaluable conceptual schemas and empirical insights on health-related stigma, and may aid in nursing and in the design of educational programs meant to combat health stigma.

## Introduction

This article examines cancer-related stigma among selected black-African people diagnosed and living with cancer in Zimbabwe. Zimbabwe, like other low-income countries is experiencing a double burden of communicable and non-communicable diseases, with significant numbers of cancer morbidity and mortality, hitherto associated with affluent nations. Globally, incidences of cancer continue to rise, as shown by the latest estimates by the International Agency for Research on Cancer [IARC], whereupon a total of 17.1 million (excluding non-melanoma skin cancer) new cancer cases per annum and 9.6 million deaths were recorded for the year 2018, up from 14.1 new cases and 8.2 million deaths in 2012 ( Bray *et al.*, 2018). Of note and concern is the higher share of deaths in the African (7.3%) and Asian (57.3%) regions, higher than the shares of incidence as a result of the varying distribution of cancer types and higher case morality in these regions ( Bray *et al.*, 2018). Nationally, according to the World Health Organization (WHO) 2018 country profile, non-communicable diseases accounted for 33% of all deaths in Zimbabwe, with cancers taking 7% of this share ( World Health Organisation, 2019). The same trend is also reflected in the latest national cancer statistics. Zimbabwe is a Southern African country with a human population of about 15.7 million. The latest annual report from the national cancer registry observes a continued upward trend in the year 2017, from 7,265 cases in 2016 to 7,659 new malignant cases recorded for the year 2017 comprising 3,270 (42.7%) males and 4,389 (57.3%) females ( Chokunonga *et al.*, 2020). A total of 2,804 deaths were recorded in the three major cities of Harare, Bulawayo and Chitungwiza, with the leading causes of death being cervical cancer (13%), followed by prostate cancer (10%), then breast (8%), oesophagus (7%), liver (6%), stomach (5%), Non-Hodgkin lymphoma (5%), Kaposi Sarcoma (4%) and colorectal (4%) in the year 2017, up from 2,751 in 2016 ( Chokunonga *et al.,* 2020). These numbers are significant given our modest total population of 15.7 million people, and given that some cases of malignancy and cancer deaths go unreported and are not captured in official statistics. Suffice to say any cost to human life is significant.

The dominant scholarship and research on cancer in Zimbabwe tends to be quantitative and is commonly undertaken within clinical and biomedical research. Such quantitative studies include trends on cancer incidence in the African population of Harare ( Chokunonga *et al.*, 2000); cancer survival in a Southern African Urban Population ( Gondos *et al.*, 2004); and age-adjusted cancer survival estimates when age-specific data are sparse ( Gondos *et al.*, 2006) among other studies. There is a need for more qualitative cancer research in Zimbabwe in general, and specifically on cancer stigma. At the time of writing, authors of this study established and were not aware of any similar study that exists in the country devoted to the subject of cancer stigma from a search on existing literature, despite the fact that there is burgeoning literature on the cancer-related stigma, elsewhere. The Livestrong Report (2007) note that around the world, cancer continues to carry a significant amount of stigma. This study explored the experience of self and enacted stigma among selected Black-African people diagnosed with cancer in Harare, Zimbabwe. There is a dearth of qualitative-situated studies which puts at the centre of analysis the prevailing socio-cultural milieu and the voice of people diagnosed with cancer, on the nature and kind of stigma that they experience. It is therefore the purpose of this study to advance social science scholarship on cancer stigma, which has been prominently studied elsewhere and not in the case study- country Zimbabwe. The other purpose of the article is to locate the study in the theoretical discourse on stigma and hence make a theoretical contribution.

Since Goffman’s 1963 classic taxonomy of stigmatized conditions, health-related stigma has received a profusion of attention among social scientists and health promotion practitioners. According to Van Brakel (2006, p. 10) “notwithstanding variations in type of illness and cultures, personal and public health-related consequences of stigma are strikingly similar”. Consequently, various literature and empirical studies have highlighted the profound negative effects of health-related stigma ( Chapple *et al.*, 2004; Else-Quest *et al.*, 2009; Link & Phelan, 2001; Link & Phelan, 2006). Health-related stigma has been shown to contribute to psychological and social morbidity, an increase in stress, strain in social life, undermining of emotional well-being, lessening of life chances, undermining of personal identity and denting of economic opportunities. This, in turn, substantially negatively affects families and results in poor quality of life among individuals with ill-health ( Applebaum *et al.*, 2014; Edelen *et al.*, 2014; Else-Quest & Jackson, 2014; Else-Quest *et al.*, 2009; Fife & Wright, 2000; Knapp *et al.*, 2014; Link & Phelan, 2001; Link & Phelan, 2006). Further, health-related stigma has been shown to be associated with premature termination of treatment, non-disclosure, poor quality of care, delayed presentation to health care professionals, amplification of psychological and social morbidity, and loss of personal control ( Chapple *et al.*, 2004; Hamann *et al.*, 2014; Knapp *et al.*, 2014; Link *et al.*, 1997; Van Brakel, 2006; Wassenaar *et al.*, 2007). Fife & Wright (2000) point to evidence which suggests that the perception of negative reactions of others determine the psychological adjustment to illness by individuals.

Stahly (1989, p. 3) posited that “cancer stigma is thought to be primarily driven by fear of the illness itself or the perception of a ‘just world’ and the idea that “it could also happen to me”. Sontag (1978) described the negative images, myths and perceived or existing physical limitations, partly explaining why illnesses are stigmatized. Consequently, the moral blameworthiness dimension, which Goffman (1963) theorized as blemished of character, is worth our scrutiny in the stigma matrix. According to Else-Quest & Jackson (2014, p. 176) “...although *general* cancer stigma has been mitigated over recent decades by increased knowledge about the disease, *specific* cancers with seemingly controllable, behavioural causes are distinctly stigmatized as self-induced or deserved”. Edelen *et al.* (2014, p. 1) note that “specific types of cancer may also carry disease-specific stigma”. For instance, regardless of the actual disease pathway, lung and cervical cancer are often in the spotlight because each is linked to behaviour that may be deemed undesirable or marginal ( Cataldo *et al.*, 2012; Chambers *et al.*, 2012; Chapple *et al.*, 2004).

### Phenomenology and cancer stigma: a cultural theoretical orientation

The use of a theoretical orientation in this study is aptly captured in the following statement,

“Theory focus on our conceptual and linguistic equipment - the nature of the location from which we look at the social world, the lexicon and syntax by means of which we talk about it, the nature of conceptual scheme, the categories into which we group things, and the logical relations that can be between concepts” ( Abend, 2008, p. 179).

Stigma is a complex phenomenon which has been variously conceptualised and reconceptualised. It was originally conceived as when a “society labels someone as tainted and less desirable on the basis of an attribute that marks them as different” ( Goffman, 1963, p. 1). Since Goffman (1963), literature has become replete with attempts to revise and reconstruct definitions of stigma, to illuminate context-specific and illness-specific elements which capture lived realities of people. This study followed in this revisionist tradition, and notable works espousing this revisionist slant include but are not exclusive to, Link & Phelan (2001), Link & Phelan (2006), Knapp *et al.* (2014), Kleinman & Hall-Clifford (2009), Major & O'brien (2005) and Carnevale (2007). Some revisionist scholars have criticized classic definitions of stigma as too individualistic, narrow and limited to micro-level analysis while giving a lacklustre attention to structural elements ( Fiske, 1998; Link & Phelan, 2001; Link & Phelan, 2006). Knapp *et al.* (2014, p 2.) followed this revisionist tradition, by spurning generalised conception of stigma experiences in favour of examining factors surrounding cancer such as type, visibility, and the likelihood that the disease will interfere with each individual’s abilities or function in a particular social context.

A prudent position on the stigma debates is proposed by Link & Phelan (2001, p. 365) when they posited that as researchers, we approach the notion of stigma from diverse theoretical perspectives that determine what gets included and what gets excluded as data. Thus, different investigators can conceptualize stigma differently, based on their epistemological grounding and as long as the meaning is explicit. Investigators who are detached from the stigmatized, tend to otherize stigmatized groups — *us versus them* and, in the course, uncritically introduce preconceived and pre-set definitions and constructs which are uninformed by study participants, but by their theoretical inclinations ( Kleinman & Hall-Clifford, 2009).

Drawing from the foregoing, by way of theoretical/conceptual framework, there are two concepts drawn from literature on health stigma which guide this study, namely self-stigma and enacted stigma. Self-stigma (also known as internalised/felt/perceived stigma) captures the tendency by the afflicted individuals to internalize societal stigma scripts and feelings of shame, fear and vulnerability ( Eba, 2007). Enacted stigma spells out actual discrimination— actions, perceptions and behaviours by the public, directed at people living with an illness ( Eba, 2007). Following Giddens’ (1984) *Structuration theory*, self-stigma and enacted stigma are a duality which cannot be separated, as the two mirror and are a reflection of each other. The structure-agency theories of figures like Anthony Giddens and Pierre Bourdieu rejects the idea of a rift between the individual (agency) and society (structure) while promoting duality of the two. Focus on both internalised and externalized manifestation of stigma at both conceptual and ontological levels is acknowledged and emphasized in recent literature ( Edelen *et al.*, 2014, p. 1). A related idea which buttresses the interconnectedness of self and enacted stigma was expressed by MacDonald & Anderson (1984, p. 285) who posited that “stigma is the result of interaction between the individual and community values”.

Further, Schulte (2002, p. 81) contrasted two models or theories about disease-related stigma drawn from his own reading of literature on stigma, namely the behavioural model and the cultural conflict model. The behavioural model proposes that stigma arises from the actions of the stigmatized and envisage consensus on defining stigma amongst observers. On the other hand, the cultural conflict model views stigma as a social construction and predicts differences in interpreting stigma behaviour among observers ( Schulte, 2002). The cultural conflict model seeks to account for stigma in line with the observer’s alliance with the prevailing cultural beliefs, and stigma variations ( Schulte, 2002). In essence these two theoretical models are invaluable to our examination of stigma in this study in that they capture both the internalization of stigma and externalization of self-stigma by individuals.

Summarily, interpretive phenomenological sociology, which inspires this study, exhorts us to blend our own perspectives and interpretations of stigma with those of research participants, in a typical fashion of ‘double hermeneutics’ ( Kumar, 2012, p. 795). Interpretive phenomenological sociology gives central concern to the *situatedness* of research participants in relation to their location in the broader socio-cultural and economic-political context. In other words, one cannot study people in isolation of their culture, family traditions, community values, or the historical period in which they live ( Mapp, 2008; Moran, 2002). Interpretive phenomenological sociology as both a theory and a method orients us to examine indices of stigma as a culmination of personality attributes of participants, and in particular socio-cultural circumstances, while weighing in the impacts of the prevailing environmental factors. This viewpoint finds expression throughout this article.

## Methods

This article is drawn from a wider phenomenological design which focused on the lived experiences of 20 people diagnosed with cancer. While participants were from different rural and urban locations, they were identified in Harare’s quaternary level of health care. Note the demographic characteristics of the participants that are presented on Table 1.

**Table 1.** Selected Sociodemographic Characteristics of the participants (n = 20).

Characteristics	Frequency	Percentage
**Age in years**		
20s–30s	5	25
40s–50s	8	40
60 and above	7	35
**Gender**		
Male	4	20
Female	16	80
**Location of Cancer**		
Cervical	6	30
Prostate	2	10
Leg	3	15
Gastrointestinal (colon, oesophagus and thyroid)	4	20
Breast	5	25

In order to enhance the heterogeneity of the sample and enhance external validity, participants diagnosed with different cancers at different time periods were recruited from the sampling frame. All participants were purposively sampled using the following criterion: a) be diagnosed with cancer from a medical institution (via biopsy); be a consenting adult (18 years and above, either male or female gender). The wider study involved a total of 30 core participants comprising people diagnosed and living with cancer while for the purpose of this article data from 20 of the 30 core participants are presented. Data from the 20 participants were pigeonholed specifically for this article as information-rich cases which reported experiences of stigma, both internalised and external. Moreover, the 20 cases embodied data saturation on the reported phenomenon of stigma and stigmatization, such that no new data was coming out from the remaining 10 cases. Principles of data saturation and disaggregation of data according to demographic characteristics along age, gender, geographical location/residence as well as part of the body where cancer was diagnosed, were catered for to enhance heterogeneity of the final sample as well as credibility, dependability, conformability and transferability. The recruitment of the 20 participants was distributed as follows: four participants snowballed from a traditional health practitioner (herbalist/faith healer) who had been attending to people diagnosed with cancer for the previous seven years; three from a register of a cancer service organisation, i.e. Cancer Centre; five from a register of a Hospice centre in Harare, while five were recruited from a register of cancer patients attending review clinics at the Radiotherapy Centre at Parirenyatwa Group of Hospitals, which is the major cancer referral centre in the country. In all the cases initial contact was done via telephone using the contact data provided, where appointments for face-to-face interviews were made with the help of personnel from the institutions.

Data were collected from May 2014 to December 2014. The article draws from a particular study objective from the broader research study, that is, to *describe and interpret lived illness experiences of selected people diagnosed with cancer in rural and urban Zimbabwe*. It is important to highlight that the corresponding author, who at the time of the field study was a holder of a Master’s degree in Sociology and Social Anthropology and male lecturer in Medical Sociology and Social Theory with the University of Zimbabwe since year 2009, conducted all the interviews in both local Shona and English languages spoken by participants. No pilot testing of any of the research tools was undertaken, in order to avoid much interference of prior knowledge, and in line with the principles of interpretive phenomenology. In addition, no data transcripts in both FGDs and interviews were returned to participants either for correction or confirmation, as both field notes and audio recordings complemented each other in cross-checking any gaps, while second or third interview sessions helped with further clarifications and probing. The corresponding author also carried out audio-taping and extensive note taking. He fully transcribed all the interviews later in the day after the actual conversations.

Approval to carry out the study was granted by the Medical Research Council of Zimbabwe (MRCZ/1834). Participants who agreed to take part signed consent forms, and pseudonyms were used instead of real names to guarantee confidentiality. Participants diagnosed with cancer were exempt from the data gathering phase during times they were sick or when in pain. Participants were free to withdraw from taking part in the study at any point in time. All interview sessions were conducted in the presence of the researcher and the participant alone except a few cases of married participants who would later request the presence of their spouse to help refresh their memory or corroborate certain experiences.

There was no existing relationship established between the researcher and participants prior to the commencement of the study. All participants were introduced to the study and the researcher through consent forms which spelt out in greater detail the identity of the researcher, the purpose of the study, why participants were selected to take part in the study as well as rights due to them if they consented to participate. Suffice to say, an intimate study of people living with cancer engendered from qualitative research, inevitably lead to long lasting contacts with participants to this day, a relationship which did not have an impact on the credibility of data. The relationship is characterised by constant updates on health matters including news of deaths of some participants from family members as well as referring newly diagnosed patients for counselling and advice by survivors (research participants). It could follow though that the introduction of the researcher as a university doctoral student could impact some responses of participants especially with regards to questions on personal knowledge of cancer and interpretation of experiences. The tendency then is for participants to shun or limit unproven/non-scientific knowledge by assuming that such knowledge will not make sense to a university researcher compared to participants’ highest education qualifications. To counter such possible interviewer bias, the researcher, grounded in phenomenological sociology, made in clear to participants that he was privileged to listen and document their unique rich experiences of living with cancer, data and information which cannot be related in textbook knowledge. In the end, participants saw the research as a platform for them to share their experiences which they felt most people who do not have cancer, have no interest to listen to. 

Conceptualization of cancer in this study was grounded in everyday expressions of participants who do not view cancer as a group of diseases as contained in biomedical journals, but as a single illness distinguished by where it develops in the human body. In essence, this entails espousing the major subject matter of interpretive hermeneutic phenomenology as a study of the lived experiences and being-in-the-world ( *Dasein*) following Martin Heidegger. Thus, the sample comprised people diagnosed with cancer on different body parts. Data collection methods (in-depth interviews, focus group discussions [FGDs], semi-detached observations and key informant interviews) were triangulated in order to address the weakness of each method as well as to cross check data from the participants. A semi-structured in-depth interview guide, focus group discussion guide, key informant interview guide and observational guide were used for collection.

In line with phenomenological inquiry, semi-structured interview sessions were conducted with each participant to gather stigma experiences of the twenty (20) participants diagnosed with cancer. A semi structured in-depth interview guide with open-ended questions was used to solicit the information. The nature of the open ended questioning ensured data saturation was reached, as the researchers had to carefully connect the pieces of the data from each interviews session and from observations as part of both data collection and preliminary interpretation of data. The first interview sessions were conducted at the quaternary health centres after a review clinic in a secure room while follow up interviews were done at homes of interviewees. The guiding question for in-depth interviews with participants diagnosed with cancer was: *Can you relate to me any experiences of stigma and discrimination that you were confronted to due to your cancer diagnosis?* There were no repeat interviews conducted. Between two and three interview sessions were conducted with each of the 20 participants, with each session lasting between 40 and 60 minutes. Further probing and prodding of issues was done in order to get thorough descriptions of stigma experiences. Field notes were made on striking responses and observable cues which were instructive to contextualisation of narratives. Participants were invited to relate stigma experiences with family members, friends, colleagues and medical professionals.

Two FGDs, using semi-structured and open ended FGD guide, were also used to collect data. FGDs were undertaken to solicit the community’s shared perceptions regarding the stigma experienced by cancer patients. The guiding question for the two FGDs conducted was: *What stigma experiences do people living with cancer that you have interacted with report?* One FGD comprised of 12 participants, including an equal number of male and female individuals was conducted in rural Chiweshe at Howard Mission Hospital (in a room provided by the Community Health Officer), approximately 96 kilometres West of the capital Harare. The participants included people diagnosed with cancer, caregivers who comprised relatives/family members, and a herbalist who joined the group at a later stage. Another FGD, comprising eight women (four of whom were diagnosed with cancer, the other four as either friends or relatives) was conducted at the home of a cancer survivor, in the middle-density suburb of Mabelreign in Harare. Each FGD lasted between 40 minutes and 1 hour, where no new data was emerging. Convenient sampling and willingness to participate were the basis for selecting the FGDs participants.

Three key informant interviews (at least two sessions) were conducted using a semi-structured and open-ended key informant interview guide. A male traditional health practitioner, a herbalist, Sekuru N.C (pseudonym) and a female oncologist were the key informant interviewees. Given that key informants interacted with the cancer patients on daily basis, their inclusion in the study provided novel stigma experiences of cancer patients they had interacted with during consultations and over the years. Key informant interviewees were purposively selected. The guiding question for the key informant interviews was: ‘ *In relation to your course of work, what are some of the major stigma experiences that have been reported by and on people living with cancer?*

Additional data was obtained from intermittent semi-detached observations carried out at Parirenyatwa Group of Hospitals, between 9 am to 12 noon on Wednesdays and Thursdays in the waiting area at Parirenyatwa Radiotherapy Center, spanning six months from May 2014 to October 2014. During those midweek days, old and new ‘patients’ diagnosed with cancer would be attending review clinics. Observations were also carried out at outdoor gardens just outside the rooms, where caregivers and patients relax before or after radiotherapy/chemotherapy sessions. At the site, the corresponding author observed (facial expressions, body postures and any observable cues related to the cancer disease/side effects like alopecia), and listened to formal and informal conversations and gossip among clients, friends and family caregivers regarding the stigma that cancer patients experience. 

### Data analysis

Data analysis was a team activity between the corresponding and the co-author. Note that at the time the research was conducted, the co-author (SM) was a holder of a Master’s Degree in Population Studies and a lecturer in the Department of Population studies. She was also a D.Phil candidate undertaking research on male reproductive cancer from a socio-cultural perspective.

Thematic analysis guided by Braun & Clarke (2006) was employed to interpret and discuss the data. Explicit reports of stigma incidences and episodes from in-depth and key informant interviews, together with generalized accounts from FGDs and semi-detached observations were read. Of note, phenomenological research essentially provides for both data gathering and data interpretation to run concurrently. Key impressions on the interpretation of data by the researcher was made during data collection, especially grounding data in its context. In essence the corresponding author exercised ‘double hermeneutics’ where interpretation of experiences by the participant was blended by the researchers’ interpretation upon reading the transcript and compilation of data stage. Therefore, implicit manifestations of self-stigma from face-to-face interviews and from observations (for example facial expressions) on people diagnosed with cancer attending clinics at the Radiotherapy Centre were inferred to note any clues for indicators of internalised stigma cited in stigma tool kits like internalised shame, depression, anxiety and low self-esteem. Compilation of data around themes was done around commonalities inherent in the narratives while discerning dominant themes or experiences. As noted by Smith (1996) the term ‘theme’ is used in phenomenology instead of ‘coding’. Themes were generated and defined after data collection. Conceptual frameworks of ‘internalised stigma’ and ‘enacted stigma’ and phenomenological theorising aided in the thematization of data. The process of data interpretation encapsulated investigators’ own reading of the data and elucidations by participants who lived the experience, in line with interpretive phenomenological sociology. In a way, thematic analysis was an attempt to make intelligible the subjective experience of illness to stakeholders in health, through thorough descriptions of novel data, noting shared similarities and individual variability ( Little *et al.*, 1998). Major and minor themes derived from dominant and minority narratives of participants were presented in the findings in line with the non-judgmental virtues of phenomenology. Direct verbatim of some participants were presented to elaborate and exemplify themes. Such a thrust has been anaemic, given the reductionist propensities of mainstream medical research which has given scant attention to embodied experience of illness ( Carnevale, 2007; Kleinman, 1988; Little *et al.,* 1998). Comparative analysis from existing literature also aided data analysis.

## Results

### Theme 1: internalised fear: dread of the disease, death and radiotherapy

One of the prominent themes that encapsulated the stigmatization of cancer in Zimbabwe revealed by this study, is the dread of the disease, emanating from the tendency to associate cancer with imminent death. The ‘news’ that someone has been diagnosed with cancer is greeted with immense trepidation in family circles and in both rural and urban communities. The current study notes that cancer is synonymized with death. A 63-year-old woman who had a mastectomy of her right breast in the year 2001, Ms V.M., recalled during an in interview her early days after the diagnosis: “ *When you hear the word cancer, eeeh. It is very scary! That word I think is what kills a lot of people as they get stress and sometimes fail to face reality*” *.* A 42-year-old woman diagnosed with cervical cancer, Mrs R.G., shared during an interview that, “ *I used to sweat in my sleep whenever I thought about my cancer*”. The fear is often deepened in light of family history of cancer and where a family member is known to have died of cancer. Notably, from the researchers’ observations at the radiotherapy centre, a familiar picture was that of distraught, sad faces moments before the arrival of doctors on duty. Of note, in a KII, a female oncologist submitted that lack of concrete assurance from health professionals to patients with cancer regarding treatment success, tended to heighten fear among patients. Further, we noted that as a result of fear and the association of cancer with death, some individuals, spouses and families chose not to disclose their condition to outsiders or even to some family members. From a FGD in Harare, a story was told of a woman with pancreatic cancer who shut the public out from visiting or talking to her, only to open up at the intervention of two women counsellors who themselves had lived for several years with cancer.

A related key finding and recurring observation from this study was a popular view shared in communities which associates hospital cancer treatment with imminent and eventual death, in a typical fashion of ‘therapeutic nihilism’. Regarding surgery, the popular belief presented from FGD data was that cancer should not be ‘disturbed’ (by way of surgery), as it was believed that any surgical operation could trigger the development and rapid progress of gangrene. Notably, the local Shona term in use for radiotherapy treatment, *kupiswa* literally translated means, ‘to be burnt’; this might in itself instil fear in most people. Health professionals bemoaned the lack of a ‘neutral’ local jargon for radiotherapy which would help to dispel fear around the treatment method (KII with oncologist). The common belief in some (rural) communities is that *ukapiswa wafa* (radiotherapy equals death). This world view is not out of sheer ignorance as indeed many had seen their friends and relatives undergo radiotherapy and die. Nonetheless, as clarified by the oncologist, some patients may be enrolled on radiation treatment only for palliative care purposes (those with advanced cancer) and not for curative purposes, and these may go on to die at any point in time. Therefore family and community members may lack the requisite knowledge on different treatment outcomes which vary according to purpose of treatment (palliative, curative) and stage of disease (KII with oncologist). Further, some community members and traditional health practitioners share the belief that once one undergoes radiotherapy, the cancer can no longer be treated successfully using traditional healing practices, or *chivanhu*. From FGDs and key informant interviews, as well as from interviews with participants diagnosed with cancer, it was noted that some traditional health practitioners discourage clients to undergo hospital cancer treatment in favour of ‘natural healing’ from traditional medicines, or divine power from God. We observe that it is imperative to differentiate stigma that emanates from the condition (disease) itself from stigma and internalised fear associated with some modern cancer treatment regimen. This distinction is worthwhile for analytical purposes though hard to pigeonhole on an ontological level.

### Theme 2: interposing culture, financial strain and self-stigma

The harsh economic environment prevailing at the time of the study, characterized by high costs of hospital cancer treatment and high levels of poverty, helps us to comprehend the internalization of stress, depression and anxiety among people diagnosed with cancer. Few previous studies on stigma bother to embrace such a holistic approach. Research findings revealed that it is not only the disease that paralyzes people living with cancer in Zimbabwe, but also, stress arising from the unavailability of financial resources needed for the next chemotherapy or radiotherapy session, or for bus fares. In an interview, a 39-year-old woman with cancer of the oesophagus, Ms S.W., reported how she would lapse into depression whenever she received a letter of warning over her unpaid bills from hospital debt collectors. The herbalist and faith healer, Sekuru N.C, shared a story of internalised stigma whereupon a woman with cancer of the oesophagus ended up refusing to take food and medicines after her relatives were now viewing her as a financial burden and *“wished to rest [die], to spare them more trouble*” (interaction of self and enacted stigma).

Further, if culture is expressed by shared norms and value codes that collectively shape a group’s beliefs, attitudes, and behaviour through their interaction in and with their environments, as noted by Airhihenbuwa (1999), the experience of the elderly diagnosed with cancer is instructive. For the elderly women from the rural areas, the prolonged nature of hospital cancer treatment alienated them from their homes, a situation which was equally distressing. From the ensuing “gossip” at the Parirenyatwa Radiotherapy Centre waiting area (semi-detached observations), it was evident that some family caregivers were constantly faced with the challenge of reasoning with their older mothers who were ready to avoid treatment or review clinics, and retire to their rural homes to oversee land cultivation and reunite with family, or *mhuri*. The older mothers were also prone to depression, which in turn triggered other health conditions like high sugar levels and high blood pressure. In Zimbabwean everyday Shona culture, a home or *musha* is of high significance to women. Thus, it is imperative to understand some contexts in which depression (one of the often-cited indicators of internalised stigma in literature) occurs among people diagnosed with cancer. In this case, stigma is felt not due to the diseases *per se* but due to playing the “sick role”. Internalised stigma is not instigated by the disease, but is impelled due to everyday living circumstances that people with cancer are confronted with, beyond the illness.

Non-disclosure is often cited as an index of self-stigma in literature ( Eba, 2007). From knowledge by hindsight, one female FGD participant in the Chiweshe area recalled how in her community, men who were diagnosed with prostate cancer and had tubes inserted (urinary catheter), kept it a secret. She recalled, “ *We used to hear about men in our communities who were said to be using tubes to pass urine and we had no knowledge that it was prostate cancer till I visited the Radiotherapy Centre*” *.* Similarly, Mrs P.M., a woman diagnosed with thyroid cancer, now familiar with the odour associated with cancer, has every reason to believe that her father possibly died of cancer of the oesophagus, although their mother hid it from them at the time. She now suspects that her cancer may be related to family genetics. Lack of knowledge of cancer combines with non-disclosure to prevent sharing of family health histories, which can assist in improving health-seeking behaviour.

Internalization of stigma can find expression in limited projection of futuristic plans by individuals (personality traits and personal circumstances such as family status and severity of illness also have an influence). Ms J.M., a widow diagnosed with cancer of the breast in 2001, narrowed her life goals to ensure she secures a good diet and gets a constant supply of medicines until she met her death, adding that she does not crave for a car or expensive clothing. When asked in an interview about his outlook for the future, Mr C.M., a 65-year-old male diagnosed with advanced cancer of the prostate, said, “ *We go through each day when it comes. When we talk about tomorrow we fool ourselves*. *We can only live a day at a time. Tomorrow may never come*”. An interesting projection of the future and the question of death were expressed by some survivors diagnosed with cancer who had recovered from earlier “ugly scenes” of the disease, thanks to successful treatment. They indicated that even if they were to die today, at least they were grateful to God that they had been given a new lease of life and had also been relieved from excruciating pain. This is notwithstanding the fear of cancer recurrence that lives with many of the people diagnosed with cancer and those on remission. The study noted that participants who were diagnosed a decade ago or more and who now attend review clinics annually or after five years, tend to be optimistic about the future, while participants who are still on chemotherapy or radiotherapy, or who have recently completed treatment, tended to be sceptical about the future or about the success of treatment.

### Theme 3: name-calling, moral blameworthiness and side effects of treatment

The study revealed enacted stigma which manifest through labelling and name-calling, which respond to external manifestations of the illness, in instances like a wound that does not heal, odour emanating from untreated persons as well as side effects of chemotherapy and/or radiotherapy. A 53-year-old woman with cancer on the leg, Mrs L.K., shared during an interview that she had been name-called by some relatives due to her unhealing wound. In a case of moral blameworthiness, a 25-year-old, single, unmarried woman with cancer of the vulva, Ms Bee, was called a promiscuous woman that “was waiting to die any time”, by her father and stepmother. In a personal interview, Ms Bee had this to say: “ *...to suffer from cancer is a very serious challenge. You lose friends and very few people accept you. ..., on one occasion my father accused me of being promiscuous and refused to meet the cost of my treatment*”.

The study revealed that side-effects of chemotherapy like alopecia in women (loss of hair), were highly stigmatized. Women participants in a FGD noted that most women put on wigs or head covers in an effort to conceal loss of hair- what Goffman (1963, pp. 41-42) referred to as “passing as normal” in order to manage “spoiled identity” *.* Ms R.G., referred to earlier in this study, admitted during an interview to “ *feeling alienated from my body, whereupon, every morning, I had to reach out for another breast (prosthesis) in the wardrobe and remove it during sleep time*”. Of note, the study established that age and marital status were salient factors in shaping stigmatization, with older women (at least 50 years old) sometimes not being bothered by the small ‘mistake’ of forgetting to put on prosthesis (artificial breasts) in public. The experience was not the same with younger married women or those who were still dating. From observations made at the Radiotherapy Centre, it was evident that younger women worried about their body image. Some of them transformed their bald heads into new-look fashion, putting on dyed, short hair, trendy earrings in a typical display of innovation and human agency.

### Theme 4: cancer stigma and straining of family relations

A persistent view revealed in this study was how cancer at times resulted in the break-up of marriages or caused severing of family relations. Some married women were distressed because of husbands who did show concern of their ill-health. All FGDs highlighted several cases of either a husband or a wife deserting an ailing partner. In one case, a husband abandoned his wife who had been diagnosed with advanced cancer of the cervix, whom he had sent to her parent’s home to be cared for. In the end, the woman had to be attended to by her brother, who was overwhelmed to the extent that when Sekuru N.C., the traditional health practitioner paid her a visit, “ *food was scattered in her room, and she was covered with soiled blankets* — *evidence of total neglect*” [words of the practitioner]. In another incident as shared by a woman participant in a FGD in Harare, a woman fled the country because her husband who had been diagnosed with cancer of the blood “ *was taking long to heal*”. Such incidences of stigma and discrimination took a toll on people with cancer, as they fully realized that “ *their loved ones were now dumping them at their point of weakness, when they needed them most”* [our own caption from FGDs].

Family ties are sometimes severed due to financial strain and the burden of care that comes with an advanced cancer. In what can be viewed as a heightened case of self-stigma, some elderly persons with cancer wished for death to spare their children the ‘trouble’ of spending their hard-earned financial resources on hospital bills (FGDs, KII). The traditional health practitioner and the oncologist cited family breakdown or strained family relations as the most pronounced, harrowing impact cancer had in communities in Zimbabwe. Nonetheless, in some cases, cancer brought some couples closer, as evidenced by the fact that not all men reported on in this study abandoned their sick wives. In an interview, Mrs P.M., a 54-year-old woman diagnosed with thyroid cancer noted that her illness had actually brought her and her husband closer. Thus, personality issues and pre-existing social relations may weigh in on stigmatization.

In the absence of institutionalized care and adequate hospital accommodation, sick people seeking treatment in the capital inevitably came to rely on extended family ties. Reminiscent of the early days of HIV and AIDS, some people diagnosed with cancer who temporarily stayed with relatives in the city, were not allowed to touch utensils such as plates, or feed a baby, due to fear that they could somehow ‘infect’ others with cancer. A 64-year-old woman (widowed) diagnosed with breast cancer, Mrs G.R., emotionally recalled during an interview some episodes of stigma and discrimination at the hands of her sister-in-law after a mastectomy, when her wound was still fresh: “ *She separated the utensils I used, for instance the bucket that I used for bathing, from items used by the rest of family. Also, she quickly disposed of my food leftovers*”. As a result, some persons with cancer would opt to sleep on the floors in public hospitals to avoid such unpleasant experiences.

Further, from experience spanning seven years of attending to many cancer patients, the herbalist narrated some harrowing observations of discrimination, isolation and rejection. He had this to say: “ *This disease, cancer, has exposed alarming anti-social behavior in our families and communities. People living with cancer are not understood. They are dumped by their relatives. They are not properly taken care of. They are rejected. Many relatives give up on them. Men, whose wives have been diagnosed with cancer, leave home and go on to remarry. As a result, most people living with cancer develop mental illness due to stress partly arising from the odour they produce, and many lose the will to live due to conspicuous rejection by their significant other*”.

The study revealed a lack of adequate knowledge on symptomatic elements of cancer as a plausible explanation for some of the discriminatory actions.

### Theme 5: unique experiences of stigma among single, young persons with cancer

The study noted that, it was a challenge to get young adults with cancer to consent to participate in our study, especially females — itself a signpost of pronounced internalised cancer stigma.

In this study, life stories of three single young adults (one male and two females) diagnosed with cancer was detailed. Single, young, dependent people diagnosed with cancer easily fall victims to accusations of immoral behaviour. A case in point was that of Ms Bee, a 25-year-old single woman with one child, who was accused by her father and stepmother of being promiscuous after she had been diagnosed with cancer of the vulva. In an interview, she remarked that, “ *...to suffer from cancer poses serious challenges. You lose friends and very few people accept you. Even your relatives think you are now a burden. For example, at one point my father told me to go and work because the bill from a herbalist who was treating me was now too high*”.

Some manifestations of self-stigma were not triggered by the illness, but by the new lifestyle one adopts as part of managing the illness. Mr S.M., a 32-year-old single male diagnosed with cancer of the colon and now living with a permanent stoma, summed up interplay of enacted and felt stigma experiences in an interview:

*This condition has really affected me emotionally such that whenever I am running out of colostomy bags l tend to have mood swings which sometimes drive me to take alcohol just to reduce worry and anxiety. I am no longer comfortable being around strangers because of the embarrassing sounds which my stomach sometimes produces. I am slowly losing confidence in dating women because I will have to explain my condition and, judging from previous experiences, everything changes the moment I disclose, which leaves me depressed. When I realized that I was never going to use the toilet the normal way; I would say that was one of my saddest moments in my whole life. The sores and stoma are a constant reminder of the suffering and pain I went through before and after the surgical operation.*

Young adult participants experience ‘social death’ when they see their chances of getting into a love relationship getting slimmer. In a sign of internalised shame and fear, a single woman, diagnosed and living with cancer of the leg, Ms V.V., noted during an interview that, “ *For us girls, no boy comes near you. You see your chances of getting into a love relationship vanish. It makes me sad*”.

## Discussion

The study examined key indicators of health-related stigma among selected people diagnosed with cancer in rural and urban Zimbabwe. The scope and substance of the study was grounded in interpretive phenomenological sociology which directs us to consider the lived experience of participants from the interpretations research participants blended with that of investigators, as well as underscoring the impact of the context or prevailing environment. The significance of cultural context in examining health-related stigma here is aptly embraced by Airhihenbuwa (1999, p. 269), who noted that, “it is the understanding of the forest that allows us to appreciate the ways in which the individual trees are shaped by the meanings constituted in the forest — the context”. Therefore, the forest is more important than the tree. Interpretive phenomenology underscores the salience of contextuality in understanding phenomena. The study found profound stigmatization experienced among selected people diagnosed with cancer in rural and urban Zimbabwe. The study also established a potpourri of stigma indicators which show a blending of internalised stigma and external stigma. The study pigeonholed five notable themes of such stigma experiences being reported (these themes were generated after data collection following key principles of data analysis of Interpretive Phenomenological Analysis). The themes include the 1) internalised fear; dread of the disease and death and dread of radiotherapy 2) interposing culture, financial strain and self-stigma 3) name-calling, moral blameworthiness and side effects of cancer treatment 4) cancer stigma and straining of family relations, incidences and episodes of blatant discrimination, rejection and isolation in homes and communities 5) unique experiences of stigma among single, young persons with cancer. All these stigma indices are an outcome of the interplay between enacted and felt stigma. Further, the prevailing economic and socio-cultural environment in the case-study country is edifying to our understanding and examination of stigma experiences as portrayed in the foregoing.

A recurring finding revealed in this study, which is confirmed by previous studies elsewhere is that cancer is a dreaded ailment, which is associated with imminent death. Previous studies have indexed fear of the disease as a primary cause of cancer stigma ( Else-Quest & Jackson, 2014; Fife & Wright, 2000; Stahly, 1989). This study showed that participants were engulfed with fear of a cancer diagnosis and after diagnosis they feared imminent death. This internalised fear is sometimes exacerbated by oncologists or health professionals who shy from giving guarantee of the success of treatment. It can be argued that the initial fear by people with cancer, which come to be internalised, can be explained by a lack of knowledge that cancer can be treated successfully if detected early. The seemingly perennial fear is also at the instigation of popular views in communities that cancer is a death sentence, which people with cancer come to internalize. Knapp *et al.* (2014, p. 5) confirm findings of this study and give credence to internalization of stigma, in their delineation of “cancer fatalism, the belief that cancer will lead inevitably to death… and this can hinder engaging in cancer prevention practices and screening, stigmatizing others, and stigmatized individuals may also internalize these attributions”. On the same note, it is imperative to note that equally dreaded among participants in this study is associated hospital treatment, notably radiotherapy, known in the local language as *kupiswa,* which literally means ‘being burnt’, as already observed. This is a key observation which needs to be taken into account when discussing cancer stigma in Zimbabwe. It may be the single most important reason why people diagnosed with cancer present late to or shun hospital treatment.

In a similar vein, Starr (as cited in Chambers *et al.*, 2012, p. 2), delineates “therapeutic nihilism as a concept ... as a belief that medical science was limited in its ability to treat disease that was considered best left to the healing powers of nature”. In such a context, innovative and aggressive health education and social marketing can be used to counter such negative thinking. This view is shared as well by Else-Quest & Jackson (2014, p. 176), who state that “better knowledge of the aetiology of cancer is critical in cancer prevention, as such knowledge can reduce fear by offering a sense of control...”

Further, the study revealed severing of family ties and straining of family relations as another key index which underlie stigma experiences of selected people diagnosed and living with cancer in Zimbabwe. This is expressed in either total rejection, blatant discrimination or breakdown of marriages among couples. People with cancer are viewed as a drain to the few financial resources available to poor families. This state of affairs likely occurs in low income and resource-constrained countries like Zimbabwe, where there are no government-initiated out-of-hospital care support systems to cater for disadvantaged people with advanced cancer. Without such support in place, people would turn to the extended family. The currently comatose economy is characterised by huge unemployment (many lack medical insurance cover provided by employers) and very low salaries against expensive cost of cancer treatment; these combine to breed high stigmatization of and on poor people with cancer in communities. If government-run out-patient care system existed, indeed this nature of stigma could be alleviated. In some cases, though, cancer actually rejuvenated social relations in a positive way.

Research findings on the nature of stigmatization experienced by young unmarried people confirmed what previous studies also found. Single, young people may have peculiar stigma experiences, informed, in part, by their age, gender, marital status, socio-economic position and family living arrangements. Eiser & Aura (2007) and Zebrack (2011) observed that the experiences of young adults diagnosed with cancer are shaped by the illness itself and the social norms and expectations associated with that stage of life. The chief concern revealed from this study is the precarious nature of love relationships, dating and prospects for marriage. Quigley (1989) elaborated that as new relationships and issues of sexuality and sterility take centre stage for unmarried survivors, disclosure becomes a daunting challenge. Moreover, stigma experiences by young adults is mediated by socio economic, gender and living arrangements which play to limit power by young people to make their own decisions on treatment and care. The end result is non-disclosure and a general fatalistic attitude from some young people with cancer. Thus, any health education programme targeting this age group should be tailor-made to address these existential concerns through therapeutic counselling and actual empowerment with economic resources.

Incidences of barefaced stigma and discrimination confronted by some people with cancer as revealed in this study, reminiscent of the early days of HIV and AIDS, serve to remind us that we cannot take for granted that many now understand disease aetiology of cancer. Previous studies have reported on the moral blame-worthiness associated with cancer of reproductive organs like the cervix, prostate and vulva as it is often linked to behaviour that may be deemed undesirable or marginal, regardless of the actual disease pathway ( Cataldo *et al.*, 2012; Chambers *et al.*, 2012; Else-Quest & Jackson, 2014). This discourse can be traced to Goffman’s (1963) theorizing of blemishes of character as a stigmatizing factor — “that having cancer reflects something morally malignant about the individual” ( Else-Quest & Jackson, 2014; Goffman, 1963). Incidences of such discrimination are underlined in name calling, moral judgements, as well as the popular belief in cancer fatalism. These expressions of discrimination may be common in most white communities in developed countries due to a longer exposure to knowledge and experience of cancer (significant cancer morbidity among black African populations in huge proportions in Zimbabwe is a fairly recent phenomenon). More targeted health education on the root causes and nature of cancer, in communities, families, churches and other cultural institutions, is essential in mitigating cancer stigma. Some best practices in health education and health promotion which were used to combat HIV- and AIDS- related stigma may be considered for cancer as well.

It is instructive to note that the presentation and analysis of cancer-related stigma in this article is typical of a localized, situated, and context-specific study, underlined by a penchant for emphasizing social construction of knowledge and reality. Kleinman & Hall-Clifford (2009, p. 418) noted that “understanding the unique social and cultural processes that create stigma in the lived worlds of the stigmatised should be the first focus of our efforts to combating and studying stigma”. A consistent argument made in this article is that at an ontological (practical) level, it becomes superficial reality to report experiences of self-stigma independent of enacted stigma. The two play out in a situation where self-stigma is a reflection of enacted stigma, as much as enacted stigma is given meaning in self-stigma. This is a key theoretical statement buttressed in light of the key study findings and is confirmed by Edelen *et al.* (2014, p. 1) who noted that “the framing of health-related stigma has begun to advance a more complex discussion of stigma, one that encompasses both the internalization of stigma by the individual and the public reaction and potential marginalization that may occur”.

 More so, accounts of stigma revealed in this study mainly apply to individuals participating, though it is prudent to assume or predict the same for individuals in similar circumstances in our Zimbabwean communities or elsewhere. Investigators oftentimes present and report cases of stigma out of the context within which they play out. In certain contexts, stigma experiences need to be read as ‘incidences’, ‘episodes’, ‘events’, ‘processes’ (temporal) and, in certain instances, as taking habitualized, enduring character (like the dread of cancer). This is a key signpost to researchers and health planners alike. Another key argument is that it is imperative to distinguish stigma which does not emanate from the disease (cancer) and stigma which is magnified and instigated by the constraining socio-economic environment. This was the case in this study, when some participants would lapse into depression due to lack of finances. It is more informing when the distinction is made, though researchers often uncritically classify depression as internalised stigma traced from the disease in question. Stigma experiences are expressed in multi-dimensional and multiple ways. A pertinent question in dissecting health-related stigma is whether one gives primacy to research participants’ interpretation or own interpretations. It is prudent to consider a juxtaposition of investigators’ and participants’ interpretation of reality.

Additionally, the over-romanticised view which needs debunking is that stigma is overly negative and that the stigmatized are *victims*, *passive actors* and *helpless*. Of note, some personal interviews with women confirmed that, “...breast reconstruction or replacement reinforces the sexual objectification of women and the felt stigma of breast cancer and mastectomy” ( Else-Quest & Jackson, 2014, p. 168). In contrast, it can be argued that most of what has been pigeonholed as internalised or felt stigma in literature may in fact constitute differential mobilization and feisty efforts by stigmatized populations to manage the debilitating impact of stigma. Some people with cancer not only fight the negative views emanating from felt or external stigma, but also actively participate in productive activities like advocacy and counselling for the newly-diagnosed. Thus, some behaviours and actions which have been pigeonholed as manifestations of self-stigma in literature and stigma tool kits (for instance, social withdrawal, non-disclosure, isolation, self-blame) may in fact point to feisty mobilization of tact and ingenuity by the stigmatized in managing their stigmatization. Nonetheless, it is not always the case that these individual management strategies succeed as Briggs *et al.* (1977) noted for example that people who live with a colostomy are not always successful in passing as “normal”, as the bags can burst, or produce odour and sound.

### Strengths and limitations of the study

The small sample size limits generalizability of findings to a wider population. However, as a qualitative study whose primary purpose was to capture the intimate expressions of stigma among selected participants, generalizability was not an evaluation criterion sought by the study. However, data from group experiences as gathered from focus group discussions, key informant interviews and observations have the potential to highlight common experiences and occurrences which can be found in communities in Zimbabwe and elsewhere. Furthermore, numerous data sources could be construed to strengthen data analysis. However, the advantages of triangulating data sources far outweighed possible flaws as this enabled the researchers to check and cross-check the validity or accuracy of data from different sources. Additionally, scientific rigor was enhanced by making data interpretation a team activity.

## Conclusions

The present study found that stigma is pronounced among people diagnosed with cancer in Zimbabwe. Its manifestation reflects both the internalization of fear and shame associated with the illness, as well as enacted elements which are tantamount to discrimination. Such a discussion which embraces both the internalization and enactment of cancer stigma and also elucidates the different indeterminate sources or manifestations of stigma, should be considered in our efforts to combat and study cancer stigma. Further, evidence discussed in the foregoing confirms the generalized form of cancer stigma and the process of stigmatization, reported elsewhere in literature as well as the unique experiences of the situated study participants. The article also demonstrated the prudence of delineating the various forms of stigma — stigma that emanates from the disease itself; stigma that is associated with treatment options; stigma that is underwritten in norms, values and everyday culture; and stigma which arises from the adoption of a new lifestyle as a result of a cancer diagnosis — thus, a positive contribution to theoretical literature. It is recommended that more targeted health education on basic cancer aetiology and associated symptoms, and knowledge explaining available hospital treatment regimens be intensified in Zimbabwe and African communities, to dispel misconceptions, miscommunication and stereotypes. In the same efforts, positive behaviours rooted in cultures may be harnessed for the same cause. Governments in resource-constrained and low-income countries are encouraged to make efforts to put in place out-patient support and care policies, as well as systems for terminal conditions like advanced cancers. This will be aimed at reducing stigma that is given impetus in an environment of scarce resources.

## Data availability

### Underlying data

The Medical Research Council of Zimbabwe does not allow the transcript data from the in-depth interviews or the focus group discussions to be made publicly available, due to the fact that policy for data circulation has to be signed. Interested researchers should contact the corresponding author or mrcz@mrcz.org.zw. Data access will be granted under the following conditions: (i) signing the data sharing agreement; (ii) waiver of the informed consents by the review board if the justifications are considered to be ethically and scientifically sound, since the consent forms clearly stated that the participants’ data would not be shared without the consent of participants.

### Extended data:

This project contains the following extended data:

Semi-structured interview guide with cancer patients.

Key informant interview Guide with Oncologist and traditional health practitioner

Focus Group Discussion Guide: (1) with cancer patients (2) a mixed group of cancer patients, caregivers and health workers.
