# Supplementary material for: A phenomenological study on experiences of cancer stigma amongst selected people living with cancer in rural and urban Zimbabwe
Source: AAS Open Res. 2021 Oct 7;4:48. [Version 1] doi: 10.12688/aasopenres.13282.1 (PMC8649965; doi:10.12688/aasopenres.13282.1)
Supplement: Peer review history

# Peer review: Reviewer response for version 1

**DOI:** 10.21956/aasopenres.14406.r28934

**Published:** 2021-12-06

**Authors:** Agostino M. Zamberia

**Affiliations:** 1 Department of Sociology and Social Work, University of Eswatini, Kwaluseni, Swaziland

This article examines the experiences of black-African people diagnosed and living with cancer in Zimbabwe, focusing specifically on cancer-related stigma. The literature reviewed in the introductory section of the article demonstrates the authors’ familiarity with the existing body of knowledge, and succinctly pulls together and integrates the arguments from previous studies and then demonstrates how the existing literature ties in with the goals of the article. Theoretical debates on stigma and stigma experiences are also adequately discussed although in their elaboration of self-stigma and enacted stigma as a duality, the authors should elaborate further on Giddens’ view that structure is both constraining and enabling.

 With regard to methodology, the study investigated the experiences of cancer stigma among twenty selected participants using interpretive phenomenological analysis derived from the methodological insights of Martin Heidegger. Accordingly, semi-structured interviews were conducted with the selected participants. Focus Group Discussions (FGDs) were also conducted to gain insights into the community’s shared perceptions relating to the stigma experienced by cancer patients. The sampling and recruitment of participants as well as the data collection strategies are clearly explained.  Approaches to data analysis, including the generation and definition of themes, and the presentation of results under the five themes identified are also adequately explained. The thematic analysis is effectively used in the development of specific arguments relating to each of the five themes. Guided by the study's methodological approach, the results section of the article provides detailed thematic analysis, offering analytical insights that inform the Discussion and Conclusions sections of the paper.

 Under Theme 4 (p.9), the authors should fact-check the statement which reads: “Some married women were distressed because of husbands who did show concern of their ill-health”. Should this read as “who did show” or “who did not show”? They should also check the veracity of the assertion in the Discussion section (p.11) that “These expressions of discrimination may be common in most white communities in developed countries due to a longer exposure to knowledge and experience of cancer.”

Is the work clearly and accurately presented and does it cite the current literature?

Yes

If applicable, is the statistical analysis and its interpretation appropriate?

Not applicable

Are all the source data underlying the results available to ensure full reproducibility?

Yes

Is the study design appropriate and is the work technically sound?

Yes

Are the conclusions drawn adequately supported by the results?

Yes

Are sufficient details of methods and analysis provided to allow replication by others?

Yes

Reviewer Expertise:

My primary research interests are in the areas of society and health, in general, and the sociology of health and illness, in particular.

I confirm that I have read this submission and believe that I have an appropriate level of expertise to confirm that it is of an acceptable scientific standard.

# Peer review: Reviewer response for version 1

**DOI:** 10.21956/aasopenres.14406.r28936

**Published:** 2021-11-01

**Authors:** Dintle Molosiwa

**Affiliations:** 1 Department of Family Medicine and Public Health, Faculty of Medicine, University of Botswana, Gaborone, Botswana

This article explores experiences of stigma among people living with cancer using an interpretive phenomenological analysis. It sought to describe and interpret lived illness experiences of selected people diagnosed with cancer in rural and urban Zimbabwe. It is a topical study that concerns an area of research that is of primary interest in Zimbabwe, Africa and beyond. The authors have provided sufficient literature review which has also clearly defined the theoretical underpinnings of the inquiry, and where the present study is likely to be of added value. However, the authors could further strengthen the literature review by providing additional background around disease and illness within the Zimbabwean socio-cultural context and in relation to stigma in general. There would be studies on stigma in relation to HIV for instance as well as mental health perhaps that the authors could draw some background framing from.~

 The authors are also encouraged to cross check their discussion section, where there are some small instances where some findings that were not indicated in the results section are referred to in the discussion section, e.g. experiences of stigma experiences by young people leading to 'general fatalistic attitude' or is that used in similar way as "social death"? Clarity and linkage will help.

Is the work clearly and accurately presented and does it cite the current literature?

Yes

If applicable, is the statistical analysis and its interpretation appropriate?

Not applicable

Are all the source data underlying the results available to ensure full reproducibility?

Yes

Is the study design appropriate and is the work technically sound?

Yes

Are the conclusions drawn adequately supported by the results?

Yes

Are sufficient details of methods and analysis provided to allow replication by others?

Yes

Reviewer Expertise:

My areas of research include health policy and systems research focusing on service delivery and especially using qualitative research methods.

I confirm that I have read this submission and believe that I have an appropriate level of expertise to confirm that it is of an acceptable scientific standard.
